# Genomic Epidemiology of SARS-CoV-2 Divulge B.1, B.1.36, and B.1.1.7 as the Most Dominant Lineages in First, Second, and Third Wave of SARS-CoV-2 Infections in Pakistan

**DOI:** 10.3390/microorganisms9122609

**Published:** 2021-12-17

**Authors:** Atia Basheer, Imran Zahoor

**Affiliations:** Genetics and Genomic Laboratory, Department of Animal Breeding and Genetics, University of Veterinary and Animal Sciences, Ravi Campus, Pattoki 55300, Pakistan; atia.basheer@uvas.edu.pk

**Keywords:** SARS-CoV-2, genomic epidemiology, lineages, clades, phylogenetic analysis, Pakistan

## Abstract

The present study aims to investigate the genomic variability and epidemiology of SARS-CoV-2 in Pakistan along with its role in the spread and severity of infection during the three waves of COVID-19. A total of 453 genomic sequences of Pakistani SARS-CoV-2 were retrieved from GISAID and subjected to MAFFT-based alignment and QC check which resulted in removal of 53 samples. The remaining 400 samples were subjected to Pangolin-based genomic lineage identification. And to infer our SARS-CoV-2 time-scaled and divergence phylogenetic trees, 3804 selected global reference sequences plus 400 Pakistani samples were used for the Nextstrain analysis with Wuhan/Hu-1/2019, as reference genome. Finally, maximum likelihood based phylogenetic tree was built by using the Nextstrain and coverage map was created by employing Nextclade. By using the amino acid substitutions, the maximum likelihood phylogenetic trees were developed for each wave, separately. Our results reveal the circulation of 29 lineages, belonging to following seven clades G, GH, GR, GRY, L, O, and S in the three waves. From first wave, 16 genomic lineages of SARS-CoV-2 were identified with B.1(24.7%), B.1.36(18.8%), and B.1.471(18.8%) as the most prevalent lineages respectively. The second wave data showed 18 lineages, 10 of which were overlapping with the first wave suggesting that those variants could not be contained during the first wave. In this wave, a new lineage, AE.4, was reported from Pakistan for the very first time in the world. However, B.1.36 (17.8%), B.1.36.31 (11.9%), B.1.1.7 (8.5%), and B.1.1.1 (5.9%) were the major lineages in second wave. Third wave data showed the presence of nine lineages with Alpha/B.1.1.7 (72.7%), Beta/B.1.351 (12.99%), and Delta/B.1.617.2 (10.39%) as the most predominant variants. It is suggested that these VOCs should be contained at the earliest in order to prevent any devastating outbreak of SARS-CoV-2 in the country.

## 1. Introduction

Since the initial reports of coronavirus disease 2019 (COVID-19) outbreak in Wuhan, China on 30 December 2019 [1,2], SARS-CoV-2 has been spreading worldwide and, as of 15 July 2021, there have been 188.13 million confirmed infections and 4.06 million deaths [3]. This was the reason that SARS-CoV-2 epidemic was declared a global pandemic by World Health Organization (WHO) on 11 March 2020 [4]. SARS-CoV-2 is a single-stranded positive-sense RNA virus belonging to the genus *Betacoronavirus*, and subgenus *Sarbecovirus*. The genome size of SARS-CoV-2 is approximately 30kb and its genomic structure has followed the characteristics of known genes of the coronavirus [5]. Albeit, RNA viruses are highly prone to mutations but Coronaviruses (CoVs) have an outstanding and distinctive feature of intrinsic proofreading mechanism [6]. However, CoVs encode a protein called nonstructural protein 14 (nsp14) that possesses a 3′ to 5′ exonuclease (ExoN) activity which is necessary for replication fidelity and proofreading activity [7]. Owing to the presence of large and complex genome, this proofreading mechanism is considered critical for maintaining normal functioning and fitness of CoVs. Perhaps, this is the reason that mutation rate in SARS-CoV-2 is 10-fold lesser compared with other RNA viruses. However, even then geneticists had reported a rate of 33 mutations/year in SARS-CoV-2 genome and now scientists are using these mutations to categorize different variants of this virus into different clades, lineages, and sublineages [4]. To date the SARS-CoV-2 has been divided in more than 81 lineages, on the basis of variations in its genome, which vary significantly in their transmissibility and virulence [8]. All the existing lineages including variants of concerns are the descendants of two ancestral lineages, A and B, discovered from China in the start of the pandemic. However, the European Center for Disease Prevention and Control (ECDC) has demonstrated five variants of concerns (VOCs) due to their high transmissibility, pathogenicity, and effects on the vaccine efficacy, which is why they are known as VOCs. The alpha variant (B.1.1.7) also known as Alpha VOC, was first detected in the UK in September 2020 [9]. This variant is highly variable and contains more than a dozen mutations compared with wild-type lineages. It has also been reported to have 50–70% high transmissibility [10], greater severity of the disease [11,12], and effects on the efficacy of the vaccine compared with wild-type lineage. The beta variant of concern, B.1.351, also known as 20H/501Y.V2 was identified from South Africa. It shares several mutations with B.1.1.7 and has the following major amino acid mutations, K417N, E484K, N501Y, D614G, and A701V in its spike protein. This lineage is also reported to have increased transmissibility and even have the advantage of escaping from immunity due to the presence of E484K mutation in its spike protein [13]. Likewise, B.1.617.2 lineage (Delta variant), also known as 20A/S:478K, was identified in India at the end of 2020 and was reported to have the following major spike mutations, L452R, T478K, D614G, and P681R, which are involved in enhancing its transmissibility and risk of hospitalization compared with B.1.1.7 [14,15,16]. Likewise, P.1 lineage (Gamma)**,** also known as 20J/501Y.V3, was identified from Brazil [17]. It has K417T, E484K, N501Y, D614G, and H655Y as the most noteworthy amino acid mutations which impact on its transmissibility and immunity [18]. B.1.427 and B.1.429 lineages (Epsilon)**,** also collectively known as 20C/S452R [19] are two more variants of concern which are based on several spike protein mutations, including L452R, which is associated with increased cell entry and reduced susceptibility to neutralization by convalescent and vaccine recipient plasma in vitro [20].

In Pakistan, the first two cases of COVID-19 were confirmed on 26 February 2020 and by 17 June 2020 each region of Pakistan has reported at least one confirmed case of Coronavirus disease. Pakistan has experienced three different waves of COVID-19, to date. The first nationwide COVID-19 wave started in late May 2020 and reached its peak in mid-June, when the number of newly confirmed cases and the number of daily deaths reached at its peak, and then in mid-July, it ended abruptly. The second wave of COVID-19 started in early November 2020 but the intensity of this wave was relatively low, and it mainly affected the southern part of Sindh. The country’s third wave began in mid-March 2021 and it mainly affected the Punjab and Khyber Pakhtunkhwa provinces. This wave peaked in late April 2021, and since then, the number of new cases and daily deaths have been decreasing. In Pakistan, 978,662 individuals had been infected with SARS-CoV-2 and 22,642 of them had died up until 15 June 2021 [21]. The first three cases of B.1.1.7 (UK-VOC) were reported on 26 February 2021, and perhaps this was the reason that the third wave of COVID-19 was much more severe compared to the first two waves. Hence, it is hypothesized that multiple variants of SARS-CoV-2 are present in Pakistan which is perhaps the reason for the repeated occurrence of COVID-19 epidemics in the country. However, the scarcity of genomics and epidemiologic studies on the transmission, spread, and distribution of different variants of virus in Pakistan has further hampered the control efforts in the country. Thus, the aims of the current study are to identify the lineages along with their origin, transmission, and prevalence in the country and the estimation of genetic relatedness among the variants, present in Pakistan and the world, through phylogenetic clustering by using the whole genome sequence data.

## 2. Materials and Methods

### 2.1. Collection of the Health Data of COVID-19 Patients

The data recorded on COVID-19 patients in different geographical regions of Pakistan as Diseased, Hospitalized, Deceased, and Recovered were taken from the official website of Pakistan (www.covid.gov.pk, accessed on 16 June 2021), for a period spanning from 3 January 2020 to 16 June 2021.

### 2.2. Viral Genome Sequences

In total, 453 whole genome sequences of SARS-CoV-2, reported from Pakistan, were downloaded from GISAID (https://www.gisaid.org/) on 10 July 2021.

### 2.3. Wave-Wise Categorization of SARS-CoV-2 Genome Sequences

The data were categorized into three waves on the basis of date of collection of samples. For the first wave which spanned from 1 January 2020 to 31 October 2020, 89 whole genome sequences of SARS-CoV2 were available. The second wave ranged from 1 November 2020 to 15 February 2021 and for this wave 118 samples of SARS-CoV-2 genome sequences were submitted to GISAID. For the third wave, which spanned from mid-February to the end of May 2021, a total of 246 whole genome sequences of SARS-CoV-2 were downloaded from the GISAID. 

### 2.4. Multiple Sequence Alignment of SARS-CoV-2 Genomes

The sequence alignment of 453 Pakistani samples was performed using L-INS-I alignment method implemented in MAFFT (v7.480), by setting data type as nucleic acids with gap extend penalty of 0.123 and opening penalties default settings of 1.53 [22]. The sequences with more than 50 ambiguous bases were removed from the data file in order to minimize the number of false positives. This resulted in the removal of 53 samples (3, 35, and 15 samples from 1st, 2nd, and 3rd wave respectively), and subsequently 400 samples (having maximum 30–40N) of complete genome were used finally for the analysis. The genome of SARS-CoV-2 reported from Wuhan, China, in December 2019, and available at GenBank with accession no. NC_045512.2 was used as a reference. The same method of alignment was used when sequences of each wave were aligned separately. 

### 2.5. SARS-CoV-2 Lineage Assignment

Phylogenetic Assignment of Named Global Outbreak Lineages (Pangolin) was used to describe the genomic lineages of the Pakistani SARS-CoV-2 sequences. The Pangolin tool follows the ‘Pango’ nomenclature system for classifying SARS-CoV-2 genomic sequences.

### 2.6. Construction of Phylogenetic Tree with Full Length Genomic Sequences

To infer our SARS-CoV-2 time-scaled and divergence phylogenetic tree, selected global reference sequences were used for the Nextstrain analysis as of 10 July 2021. This dataset consisted of 3804 sequences sampled between 26 December 2019 to 10 July 2021 from Africa (541), Asia (937), Europe (727), North America (779), Oceania (512), and South America (566). A collection of 400 Pakistani SARS-CoV-2 sequences was added to generate an initial dataset of 4204 whole-genome sequences. Using the Nextstrain metadata [23] to identify the accessions of interest, the latest whole-genome sequence alignment from the GISAID database was downloaded. These 4204 whole genome sequences were aligned using MAFFT (v.7.480) [22], through multiple sequence alignment method, and 20–30 bp from start and around 50 bp at the end were masked and any gap only sites and low-quality sequences were removed. The Wuhan/Hu-1/2019 (EPI_ISL_402125), sampled on December 31, 2019, from Wuhan, China was downloaded from the GISAID and used as reference genome. Finally, the maximum likelihood phylogenetic tree was built by using the Nextstrain pipelines which incorporates Augur for generation of phylogenetic tree and Auspice for visualizations. A coverage map was created using Nextclade (version 0.7.5), which performs banded Smith–Waterman alignment with an affine gap penalty.

### 2.7. Wave-Wise Constrution of Phylogenetic Tree

The phylogenetic tree of each wave was inferred by using the Maximum Likelihood method based on amino acid substitution model [24] of Jones Taylor Thornton (JTT) matrix-based model in Mega-X software. In each wave, initial phylogenetic tree(s) for the heuristic search were obtained automatically by applying Neighbor-Join and BioNJ algorithms to a matrix of pairwise distances estimated using the JTT model, and then finally, the maximum likelihood phylogenetic tree of each wave was constructed by selecting the topology with superior log likelihood value. 

## 3. Results

**Figure 1:**
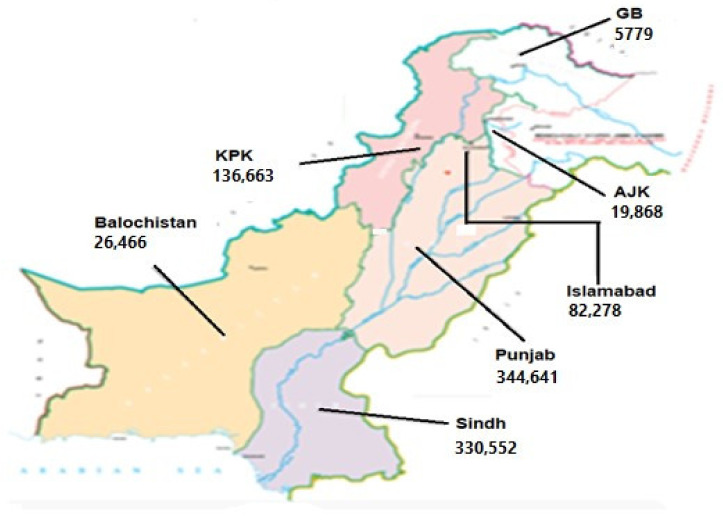
Number of the total cases of COVID-19 in different provinces/territories of Pakistan up to 16 June 2021. COVID-19 cases of Islamabad, Capital of Pakistan, are also shown here. This map is adopted from https://covid.gov.pk/ accessed on 16 June 2021.

The data about the health status of COVID-19 patients in different geographical regions of Pakistan, taken from the official website of Pakistan (www.covid.gov.pk, accessed on 16 June 2021), are presented in Table 1 and Figure 1.

It is observed that maximum COVID-19 cases were detected in Punjab (344,641) followed by Sindh (330,552), KPK (136,663), Baluchistan (26,446), AJK (19,868), Capital city of Islamabad (82,278), and Gilgit-Baltistan (5759) region of Pakistan (Figure 1). During this duration, 21,913 deaths were recorded in Pakistan, out of which the highest numbers (10,603) were observed in Punjab province.

### 3.1. Major Waves of SARS-CoV-2 Infection in Pakistan

**Figure 2:**
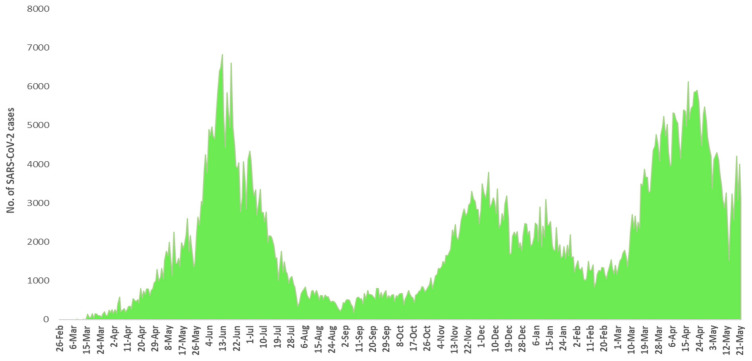
Month-wise details of SARS-CoV-2 cases starting from February 2020 to May 2021.

**Figure 3:**
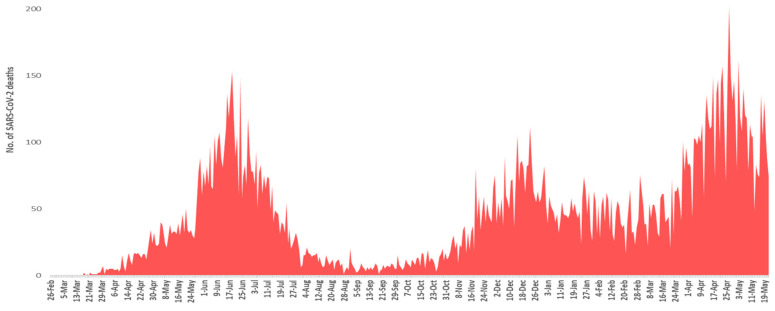
Month-wise details of deaths caused by SARS-CoV-2 starting from February 2020 to May 2021.

Pakistan has observed three major waves of SARS-CoV-2 infection. The nation’s first wave of SARS-CoV-2 began in late May 2020, peaked in mid-June and ended in mid-July. In this wave, Pakistan experienced the highest number of cases and mortalities during its peak month. However, this wave passed away very rapidly and new cases and mortalities were dropped suddenly after the peak. The second surge of COVID-19 started from the end of October 2020 to mid-February 2021. This wave was low in its intensity, mainly affecting the southern parts of Sindh province, and peaked in mid-December 2020. The country’s third wave began in mid-March 2021, when daily new confirmed cases and mortalities began to rise steeply. The third wave mainly affected the provinces of Punjab and Khyber Pakhtunkhwa. This wave peaked in late April 2021, and since then daily new cases, and new mortalities have been falling. The complete pictures of number of SARS-CoV-2 positive cases and mortalities from February 2020 to May 2021 are shown in Figure 2 and Figure 3.

### 3.2. Distribution of Genomic Lineage of SARS-CoV-2 in Pakistan

**Figure 4:**
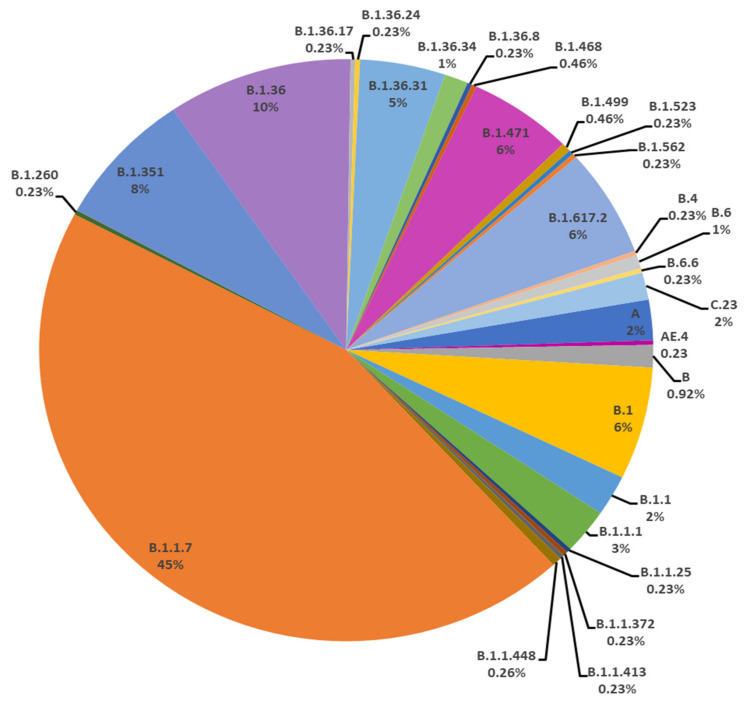
All the lineages observed from Pakistan sequences downloaded on 10 July 2021.

In the current study, a total of 453 SARS-CoV-2 genomes sequences downloaded from GISAID on 10 July 2021 were used. In Pakistan, 29 lineages were observed by using Phylogenetic Assignment of Named Global Outbreak Lineage (Pangolin 3.1.4, http://www.pangolin.cog-uk.io). In total, 53 samples were failed. The overall lineage distribution highlighted the occurrence of B.1.1.7(*n* = 178), B.1.36(*n* = 39), B.1.351(*n* = 30), B.1(*n* = 25), B.1.617.2(*n* = 24), B.1.471(*n* = 22), B.1.36.31(*n* = 18), B.1.1.1(*n* = 10), B.1.1(*n* = 9), A(*n* = 9), C.23(*n* = 6), B(*n* = 5), B.1.36.17(*n* = 1), B.1.36.34(*n* = 5), B.6(*n* = 3), B.1.499(*n* = 2), B.1.1.448(*n* = 2), AE.4(*n* = 1), B.1.260(*n* = 1), B.1.36.24(*n* = 1), B.1.36.8(*n* = 1), B.1.468(*n* = 1), B.1.523(*n* = 1), B.1.562(*n* = 1), B.1.1.25(*n* = 1), B.1.1.372(*n* = 1), B.1.1.413(*n* = 1), B.4(*n* = 1) and B.6.6(*n* = 1) as mentioned in Figure 4 and Table 2.

### 3.3. Distribution of GISAID Clades of SARS-CoV-2 in Paksitan

**Figure 5:**
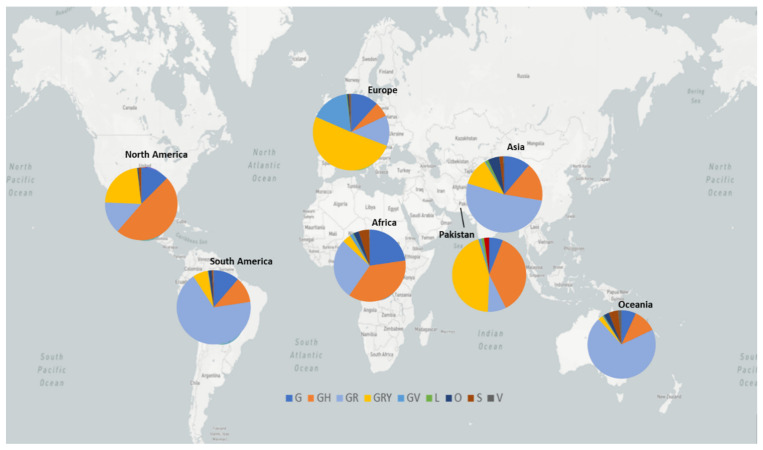
GISAID clades of SARS-COV-2 genome from global and Pakistani datasets. The map in the background is adopted from https://nextstrain.org/sars-cov-2/ accessed on 16 June 2021 [23].

The distribution of GISAID clades of SARS-CoV-2 genomes from Pakistani and global dataset, downloaded on 10 July 2021, is presented in Table 2 and Figure 5. The genomic sequences of Pakistani SARS-CoV-2 samples indicate the prevalence of 7 clades with following order of prevalence GRY (44.5%), GH (36.75%), GR (7.75%), G (6%), S (2.25%), L (1.50%), and O (1.25%).

### 3.4. Occurrence of Variants of Concerns in Pakistan

Our data also revealed the presence of the three VOCs including Alpha (B.1.17), Beta (1.351), and Delta (B.1.617.2) variants in Pakistan, with the following order of genomic prevalence 44.5%, 7.5%, and 6% respectively.

### 3.5. Phylogenetic Analysis

**Figure 6:**
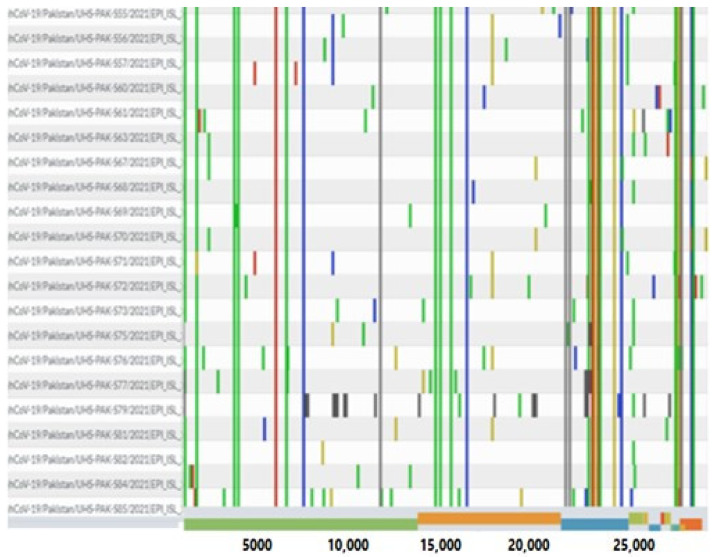
Genomic map of SARS-CoV-2 genomes of Pakistan. The reference genome in this coverage map is Wuhan/Hu-1/2019.

**Figure 7:**
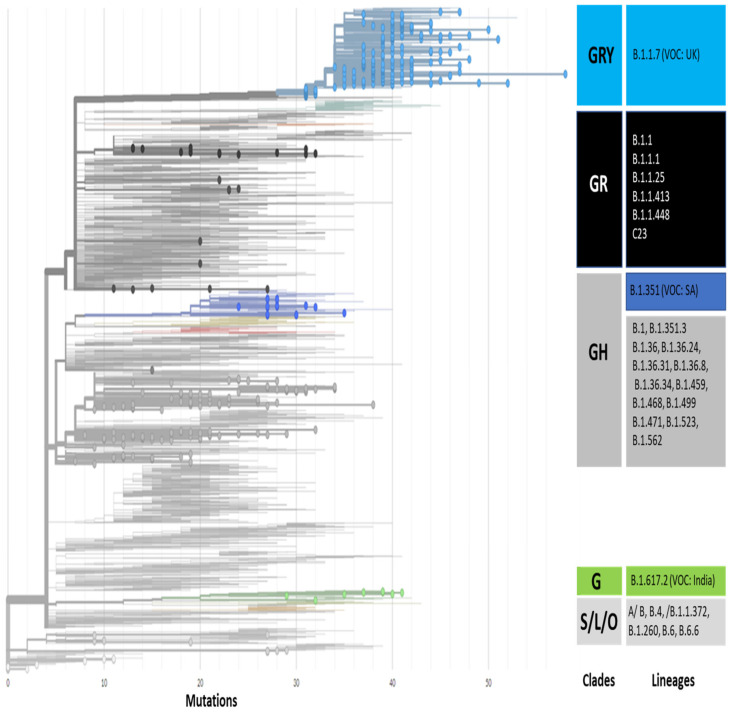
Maximum likelihood phylogenetic tree of Pakistani samples showing 7 clades and 29 lineages. The global SARS-CoV-2 genomes depicting the highest mutations ranging from 30 to 50 in Pakistani samples under the lineage of B.1.1.7, also known as UK variants of concern (UK-VOC). The light blue, dark blue, black, green, light & dark gray dots in the phylogenetic tree show the distribution of Pakistani SARS-CoV-2 samples in the respective clades.

**Figure 8:**
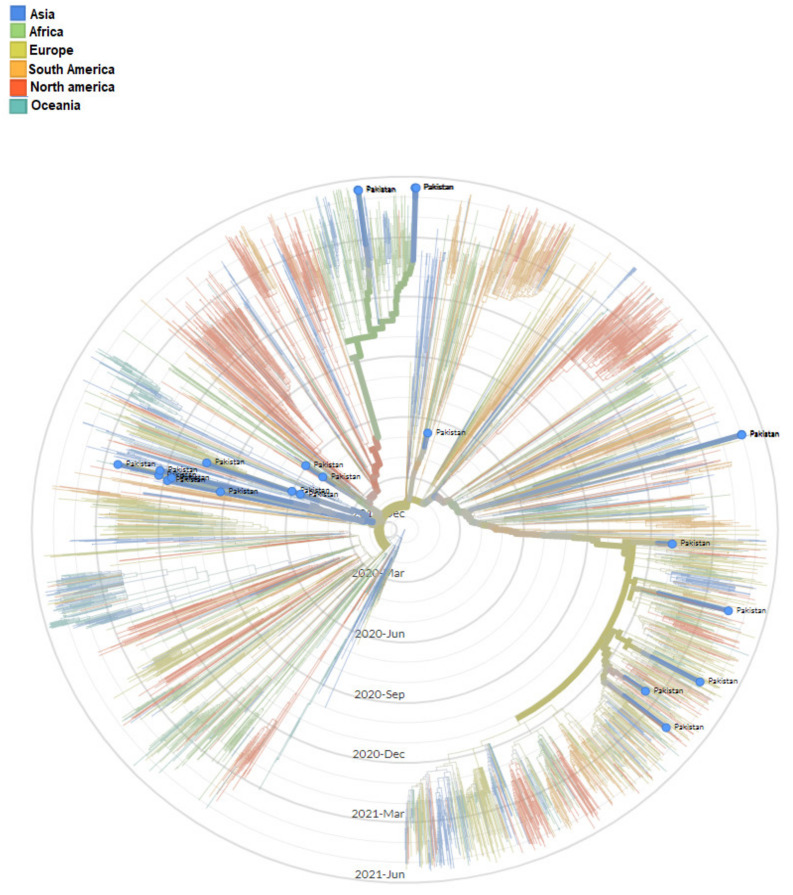
Maximum Likelihood time resolved phylogenetic tree of global samples representing the emergence of SARS-CoV-2 in Pakistan.

The genomic map of SARS-CoV-2 of Pakistani samples depicting the mutational profiles is given in Figure 6. Maximum Likelihood Phylogenetic tree of 400 whole genome sequences of Pakistani SARS-CoV-2 was developed as per definitions of the PANGOLIN lineage and GISAID clades (Figure 7). The overall clades and lineages distribution highlighted the dominant occurrence of GRY (B.1.1.7), GH (B.1, B.1.351, B.1.36, B.1.36.17, B.1.36.24, B.1.36.31, B.1.36.34, B.1.36.8, B.1.468, B.1.471, B.1.499, B.1.523, B.1.562), GR (AE.4, B.1.1, B.1.1.1, B.1.1.25, B.1.1.372, B.1.1.413, B.1.1.448, C.23), G (B.1.617.2), S (A), L (B, B.4), and O (B.1.260, B.6, B.6.6) clades respectively. The maximum likelihood time resolved phylogeny tree of Pakistani and global samples is presented in Figure 8.

### 3.6. Wave-Wise Phylogenetic Trees of SARS-CoV-2

**Figure 9:**
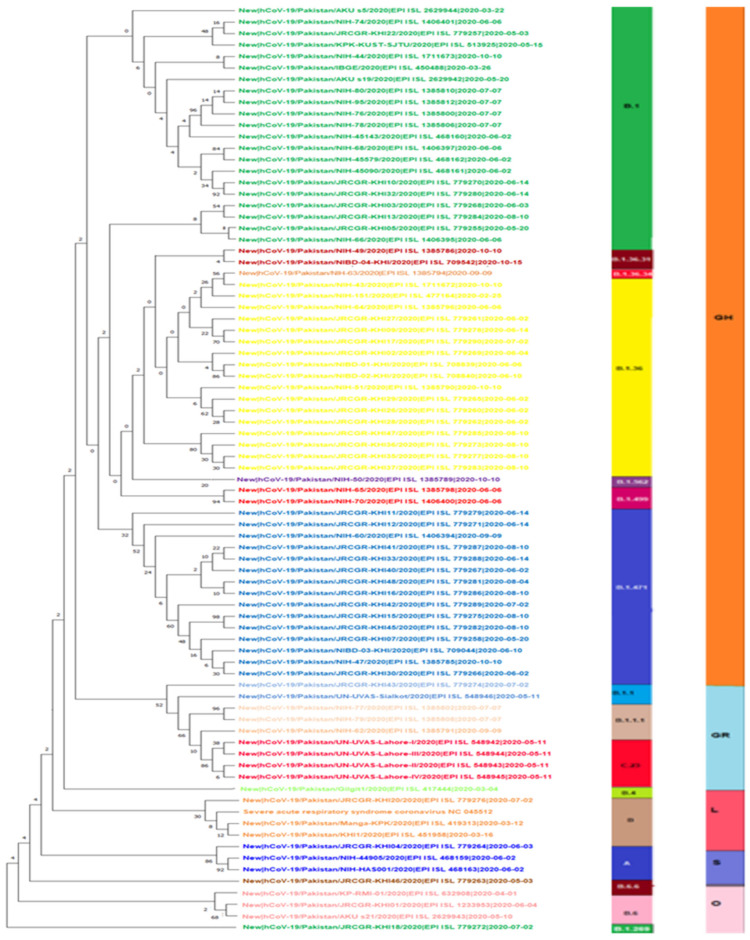
Maximum Likelihood phylogenetic tree of SARS-CoV-2 genome during first wave of infection representing 16 (A, B, B.1.1, B.1.1.1, B.1.260, B.1.36, B.1.36.31, B.1.36.34, B.1.471, B.1.499, B.1.562, B.4, B.6, B.6.6, C23) lineages of 5 clades (GH, GR, L, S, O) in first wave.

**Figure 10:**
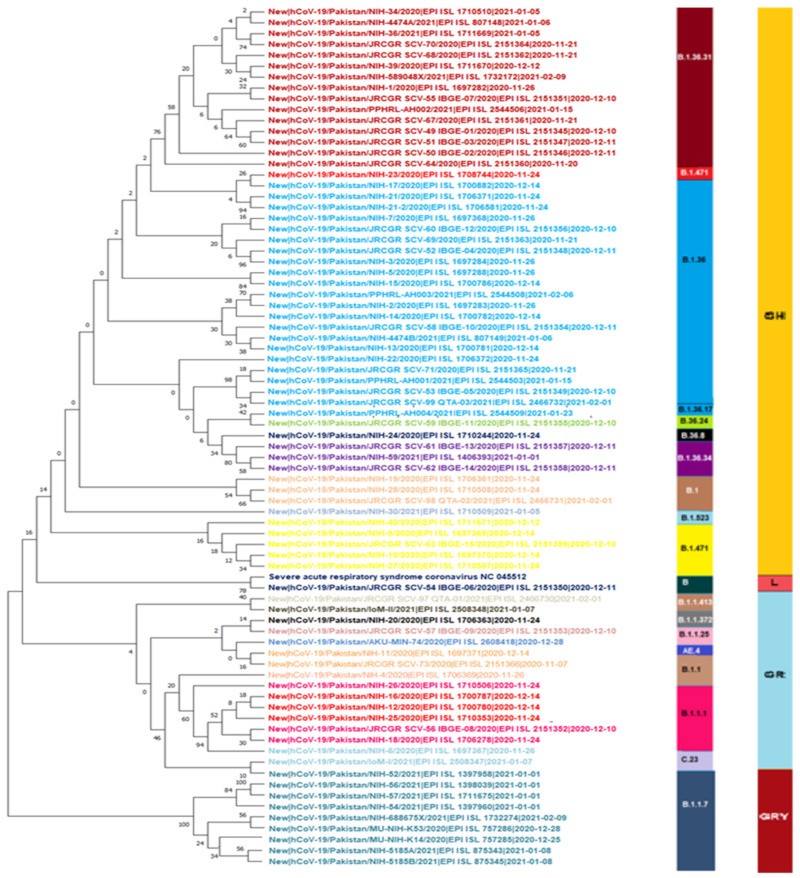
Maximum Likelihood phylogenetic tree of SARS-CoV2 genome during second wave representing 19 lineages (B.1.36.31, B.1.471, B.1.36, B.1.36.17, B.36.24, B.36.8, B. 1.36. 34, B.1, B.1.523, B.1.471, B, B.1.1.413, B.1.1.372, B.1.1.25, AE. 4, B.1.1, B.1.1.1, C.23, B.1.1.7) and 4 clades (GH, L, GR, GRY) in second wave.

**Figure 11:**
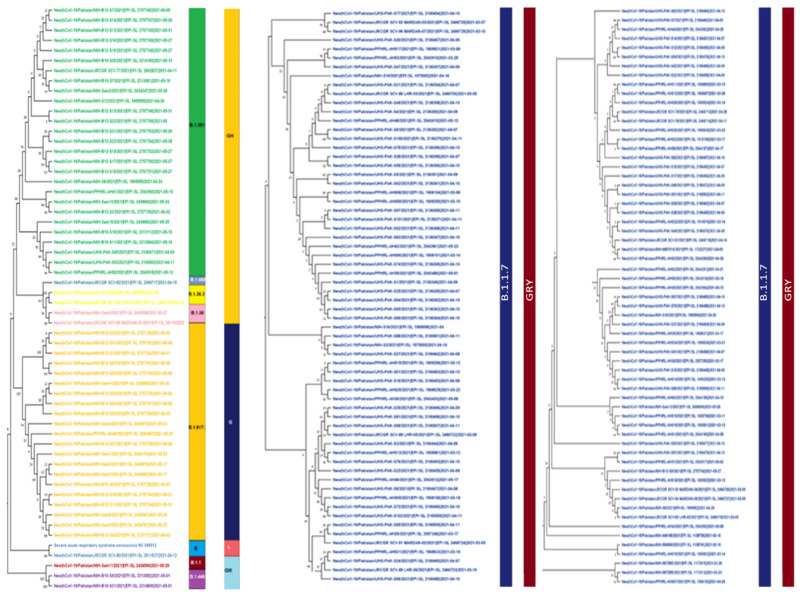
Maximum Likelihood phylogenetic tree of SARS-CoV-2 genome spitted in three columns. This tree is representing 9 lineages (B.1.351, B.1.468, B.1.36.31, B.1.36, B.1.617.2, B, B.1.1, B.1.448, B.1.1.7) and 5 clades (GH, G, L, GR, GRY) in third wave of SARS-CoV2 infection.

**Figure 12:**
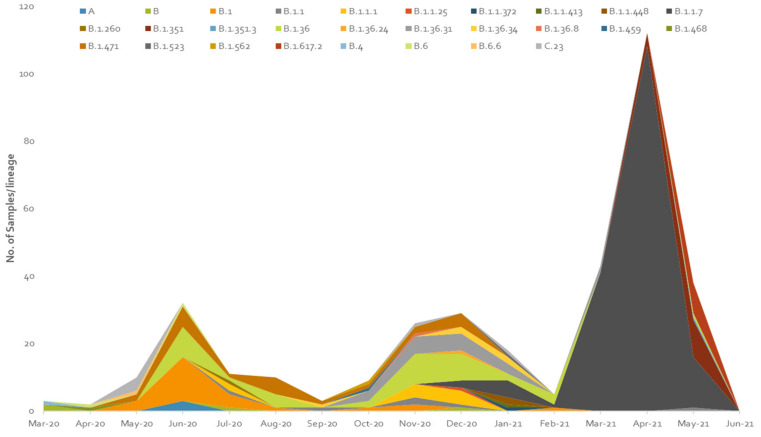
Pictorial view of genomic-data-based incidence of different lineages of SARS-CoV-2 observed during the three waves of COVID-19 in Pakistan.

The maximum likelihood phylogenetic tree of first, second, and third wave are presented in Figure 9, Figure 10 and Figure 11. The tree of the first wave showed 16 lineages of 5 clades, with GH as the major clade in this wave (Figure 9). During the second wave, 19 lineages belonging to 4 clades were observed and GH was again the major clade in this wave (Figure 10). In third waves, 9 lineages representing 5 clades were observed (Figure 11), and GRY was the major clade in this wave (Figure 12).

## 4. Discussion

The first two cases of COVID-19 were reported in Pakistan in February 2020 and since then the country has observed 978,662 infections and 22,642 deaths till 15 July 2021 [21]. However, the whole genome sequences of the first three SARS-CoV-2 samples, collected in March, revealed the presence of B and B.4 lineages in the country. The lineage B.4 was reported to have a major role in the early pandemic in Iran [25]. This supports the hypothesis of an early transmission of the virus to Pakistan most likely from Iran and China, and this is also consistent with the statement of Pakistan’s health ministry officials who confirmed the first two cases of COVID-19 on 26 February 2020 from patients who travelled back to Pakistan from Iran. This can be further confirmed by the fact that up to the first week of April 2020, 75% of COVID-19 positive patients had a history of traveling to Pakistan from Iran [26]. The occurrence of severe outbreaks of COVID-19 in two neighboring countries and declaration of the disease as a pandemic by WHO prompted the Pakistan government to close the border with China and put very strict screening methods at the Pakistan-Iran border [27,28]. By following the guidelines of Federal government, they devised “The National Action Plan for The Corona Virus Disease (COVID-19) Pakistan”, the quarantine centers were established in a few big cities including Islamabad [29,30] and along the Pakistan-Iran border to help identify positive cases and quarantine pilgrims returning to Pakistan after spending time in Iran [29]. However, even with all of these efforts, major lapses existed at every step, mainly the uneven implementation of immigrant policies to deal with the influx of people from the outside of the country [29], the lack of facilities, poor infrastructure, and apathy & indifference among people to follow government devised Corona SOPs, which subsequently led to the spread of COVID-19 throughout the country [31]. 

### 4.1. First Wave of SARS-CoV-2

Pakistan experienced three major waves of SARS-CoV-2 infection. The first wave mainly covered three months starting from May 2020 to July 2020 (Figure 2 and Figure 3). From this wave, whole genome sequences of 86 samples of SARS-CoV-2 were analyzed. These samples represented 16 lineages belonging to 5 clades (Figure 9) including A(9.3%), B(4.6%), B.1(24.4%), B.1.1(2.3%), B.1.1.1(3.4%), B.1.260(1.16%), B.1.36(18.6%), B.1.36.31(2.32%), B.1.36.34(1.2%), B.1.471(18.6%), B.1.499(2.32%), B.1.562(1.2%), B.4(1.2%), B.6(3.4%), B.6.6(1.2%), and C.23(4.65%). Lineage B.1 hit the highest score in the first wave in Pakistan and internationally this lineage was reported to have a very high incidence in North America (the highest incidence), UK, Germany, and Spain [32,33]. It is also noteworthy that the highest proportion of Pakistani diaspora is living in these countries. Therefore, it is highly likely that the transmission of this lineage to Pakistan took place through international travelers most probably through the travelling of Pakistani diaspora living in these countries. Although Pakistan suspended all international flights from 21 March to 30 May 2020, it is still highly likely that before the suspension of flight operations [34] many international passengers (positive for these lineage) managed to enter the country. This hypothesis is further augmented from the fact that almost all the sequence samples of this lineage were from big cities including Karachi, the capital city of Islamabad, and Lahore, which have international airports. The lineage for second highest incidence of infection of SARS-CoV-2 in Pakistan was B.1.36. This lineage had the highest incidence of infections in India, Canada, UK, and Hong Kong. Likewise, B.1.471 was also the second most prevalent lineage with an incidence of 18.6%. The whole genome sequence data of SARS-CoV-2 from GISAID predict the first incidence of this lineage in Saudi Arabia on 25 March 2020 [35]. After that, this lineage was observed in Pakistan suggesting the direct transmission of this lineage from Saudi Arabia to Pakistan. Likewise, the most likely reason for the transmission of this lineage to Pakistan was again through international travelers. Another possible explanation for the transmission of these lineages could be the congregation (250,000 peoples) of Tablighi Jamaat in Pakistan in which in addition to Pakistan, 3000 delegates from 40 different countries did participate, with significant proportion of delegates from India [36]. This hypothesis could be further confirmed from the timeline of the reports of these lineages in Pakistan as the congregation took place in early March 2020 and these lineages were reported for the first time in May and had the highest incidence in June 2020.

It is also worth mentioning that the first four cases of C.23 lineage were reported for the first time from Pakistan on 11 May 2020 and 3–4 weeks later this lineage was also reported from the USA. It is also highly likely that this lineage was transmitted to the USA from Pakistan through the Pakistani diaspora returning back to the USA after visiting their homeland. However only 148 cases of this lineage have been reported globally up to 15 July 2021 indicating the lesser transmissibility of this lineage.

### 4.2. Second Wave of SARS-CoV-2

The second spell of SARS-CoV-2 infection in Pakistan ranged from the end of October 2020 to mid-February 2021, however, it was at its peak from mid-November to mid-December 2020 (Figure 2 and Figure 3). For this wave, after QC analysis 35 incomplete sequences of SARS-CoV-2 were removed and the remaining 83 samples with whole genome sequences, available on GISAID, were analyzed. According to our dataset, 18 lineages belonging to 4 clades (Figure 10) were observed in this wave, out of which 10 were the same lineages which were already prevailing in the country during the first wave and 8 others were the new lineages. The B.1.36 (initially reported from India, Canada, and UK) which was the second most common (18.6%) lineage during the first wave in Pakistan, had the highest incidence in the second wave as well. Interestingly it was also observed that after the transmission of this lineage to Pakistan it remained prevalent in the country and positive cases of this lineage were observed in almost every month during the first and second wave. The B.1.36.31 (a sub-lineage of B.1.36) which was also present in the first wave had the second highest incidence in the second wave. The first case of B.1.36.31 lineage was reported from Australia in early March 2020, then two cases of this lineage were reported from England, and the fourth case was reported from Pakistan. The incidence of this lineage in the second wave remained 16.9% and out of the total 532 global genomic sequences of this lineage, 18 were reported from the Pakistan during the second wave (mostly from Karachi).

Likewise, the lineage B.1.471 which also had the second highest (18.6%) incidence during the first wave, remained the third most common lineage in the second wave but its incidence was reduced to 7.23%. It is also noteworthy that only 346 samples of this lineage were observed out of global sequence data available at GISAID, out of which 22 were reported from Pakistan only. And this lineage was also continuously observed from samples sequenced from May to December 2020, during the first and second wave of SARS-CoV-2 infection, in the country. However, B, B.1, B.1.1, and B.1.1.1, which were present in the first wave, had incidences of 1.2%, 4.82%, 7.23%, and 8.4% respectively in the second wave as well.

Among the new lineages of second wave the B.1.1.7, B.1.36.34 and C.23 had 12.1%, 4.8%, and 2.4% incidences respectively. The first case of B.1.36.34 was reported from Canada in May 2020 and (four months later) in September 2020 the genomic sequence of this variant was reported from Pakistan [35]. It is most likely that both B.1.36.31 and B.1.36.34 were transmitted to Pakistan through overseas Pakistanis, a significant number of whom live in Canada and Australia and used to travel to Pakistan frequently. However, only 41 cases of B.1.36.34 have been reported globally, till 10 July 2021, out of which 5 were reported from Pakistan (Table 2). The very small number of sequences of this lineage worldwide suggest its low transmissibility compared with other lineages. The B.1.1.7 (UK-VOC) lineage had the highest incidence in the second wave but it was reported almost at the end of the second wave and caused the highest infection rate in the third wave. 

B.1.36.17, B.1.36.24, B.1.1.25, B.1.1.372, B.1.1.413 were the minor lineages in the second wave and had their first cases reported mainly from England and Wales [35] and were also likely to be transmitted to Pakistan from UK. Likewise, B.1.36.8, B.1.523, and AE.4 had their first case reported from India, Saudi Arabia, and Bahrain respectively in early 2020 [35]. They were also likely transmitted to Pakistan via international travelers. 

### 4.3. Third Wave of SARS-CoV-2

The third wave of SARS-CoV-2 proved worst in the country with the highest number of infections (335,728) and deaths (7849) compared with the previous waves. In the third wave, B.1.1.7, B.1.617.2, and B.1.351 were the major lineages for the SARS-CoV-2 based infections (Figure 11 and Figure 12). During this wave, first two cases of B.1.1.7 variants were reported on 20 December 2020 in the country and kept spreading continuously and had the highest incidence during the peak month of third wave. This was further evidenced by the submission of 108 (60.6%) genomic sequences of B.1.1.7 (UK VOC), only in April 2021, out of the total 178 genomic sequence of this lineage from Pakistan, starting from 1 December 2020 to 10 July 2021 (Figure 4). The B.1.1.7 variant is estimated to have emerged in September 2020 and quickly became the dominant circulating variant in England [35]. In addition to Pakistan, this variant was reported from 30 other countries. Moreover, until 17 June 2020 there was no vaccine available for COVID-19, and from the spring of 2021 the vaccine was only available to those people who were over 50 years of age. However, in the case of drugs doctors used all kind of supportive treatment starting from steroidal and nonsteroidal anti-inflammatory drugs to bronchodilators g with the use of both. To treat COVID-19 some anti-malarial drugs like hydroxychloroquine and some antiviral drugs like Tocilizumab, Bemsivir lyophilized, Ninavir lyophilized, and Azithromycin, were also used as therapeutic agents [37,38]. Moreover, convalescent plasma was also used to treat the seriously affected COVID-19 patients here in Pakistan.

The modeled trajectory of this variant (B.1.1.7) in Pakistan revealed a rapid increase in cases in early 2021, making it the predominant variant in the third wave (Figure 7, Figure 8 and Figure 9). Consistent with our findings several other authors had reported that this variant had high transmissibility, virulence, and death rates compared with other variants of SARS-CoV-2 [12,39,40]. Moreover, it is also worth mentioning that this variant had very high mutation rate (ranging from 45–50) (Figure 7), in Pakistan, compared with other variants. Looking at the severity of the third wave, it is speculated that these mutations might have resulted in enhancing the transmissibility and pathogenicity of this variant in Pakistan.

B.1.617.2 lineage (Delta variant) was another variant which was observed almost at the end (May 2021) of the third wave of COVID-19 in Pakistan. It was originally reported from India in April 2020. Scientists have reported that the Delta variant is 40–60% more contagious and virulent than the B.1.1.7 [41,42] and had caused a catastrophic wave of COVID-19 in India [41]. However, Pakistan remained lucky enough that this lineage was contained very shortly and in total only 24 genomic sequences of this variant had been reported from Pakistan, till 10 July 2021. However, this variant alone is known to have caused at least four million deaths worldwide, mainly in India and the UK [42].

B.1.351 lineage (Beta variant) was also reported during the third wave in Pakistan. This variant is known as the South African variant but its first few cases were reported from England in the start of 2020 [43]. However, from Pakistan the first case of this lineage was reported in May 2021 and it is highly likely that this variant was transmitted to Pakistan from England through overseas Pakistanis. This variant is also known to have significantly greater transmissibility over Alpha-VOC [42,44,45]. However, only 30 genomic sequences of this variant have been reported so far (10 July 2021) from Pakistan which are 12.9% of total genome sequences of SARS-CoV-2 submitted from Pakistan during the third wave of COVID-19. Our data revealed that B.1.1.7, B.1.351, and B.1.617.2 were the dominant lineages in the third wave, and this is the most likely the reason for the severity of this wave in terms of infections and deaths in the country.

## Figures and Tables

**Table 1:** The data of COVID-19 taken from the official covid website (http://www.covid.gov.pk, accessed on 16 June 2021) of Pakistan for a period spanning from 3 January 2020 to 16 June 2021.

Areas	Diseased	Hospitalized	Deceased	Recovered
Azad Jammu & Kashmir (AJK)	19,868	447	571	18,850
Baluchistan	26,446	794	300	25,372
Gilgit-Baltistan	5759	105	108	5546
Islamabad	82,278	1216	773	80,289
Khyber Pakhtoon Khawah (KPK)	136,663	2958	4252	129,453
Punjab	344,641	10,462	10,603	323,576
Sindh	330,552	19,827	5306	305,419

**Table 2:** List of 29 lineages of SARS-CoV-2 observed in Pakistan along with their local and global percentages.

Clade	Pakistan Lineages	No of Samples Sequences from Pakistan	Percentage of Lineages in Pakistan	No of Samples Sequenced Globally of Pakistani Lineages	Global Percentage of Pakistani Lineages
G	B.1.617.2	24	6	158,368	0.1178
GH	B.1	25	6.25	85,731	0.0638
	B.1.351	30	7.5	28,385	0.0211
	B.1.36	39	9.75	6059	0.0045
	B.1.36.17	1	0.25	2027	0.0015
	B.1.36.24	1	0.25	158	0.0001
	B.1.36.31	18	4.5	543	0.0004
	B.1.36.34	5	1.25	43	0.0000
	B.1.36.8	1	0.25	936	0.0007
	B.1.468	1	0.25	172	0.0001
	B.1.471	22	5.5	350	0.0003
	B.1.499	2	0.5	760	0.0006
	B.1.523	1	0.25	388	0.0003
	B.1.562	1	0.25	53	0.0000
GR	AE.4	1	0.25	20	0.0000
	B.1.1	9	2.25	48,427	0.0360
	B.1.1.1	10	2.5	2774	0.0021
	B.1.1.25	1	0.25	1641	0.0012
	B.1.1.372	1	0.25	1339	0.0010
	B.1.1.413	1	0.25	105	0.0001
	B.1.1.448	2	0.5	468	0.0003
	C.23	6	1.5	153	0.0001
GRY	B.1.1.7	178	44.5	994,397	0.7395
L	B	5	1.25	5918	0.0044
	B.4	1	0.25	439	0.0003
O	B.1.260	1	0.25	168	0.0001
	B.6	3	0.75	1052	0.0008
	B.6.6	1	0.25	1043	0.0008
S	A	9	2.25	2694	0.0020
Total Pakistani Sequences Analyzed		400	No. of Total Global Sequences	2,302,116	
